# Supplementary material for: Impact of Pre-Diagnostic Risk Factors on Short- and Long-Term Ovarian Cancer Survival Trajectories: A Longitudinal Observational Study
Source: Cancers (Basel). 2024 Feb 28;16(5):972. doi: 10.3390/cancers16050972 (PMC11154316; doi:10.3390/cancers16050972)
Supplement: Supplementary file 1 [file cancers-16-00972-s001.zip › cancers-2821510-supplementary.pdf]

Table S1. HR and 95% CI of risk factors and ovarian cancer-specific mortality by survival time intervals [a]

Risk Factors	N	< 3 years		3 - < 6 years		6 - < 10 years		≥ 10 years	
		Cases	HR (95% CI)	Cases	HR (95% CI)	Cases	HR (95% CI)	Cases	HR (95% CI)
**Tumour characteristics**									
Age at diagnosis, n (%)									
<50 years	390	47	0.92 (0.63, 1.35)	65	1.02 (0.74, 1.41)	34	0.85 (0.54, 1.32)	14	0.73 (0.36, 1.47)
50 to <60 years	410	63	1.00 (ref)	85	1.00 (ref)	46	1.00 (ref)	18	1.00 (ref)
60 to <70 years	348	62	1.14 (0.80, 1.62)	79	1.19 (0.88, 1.63)	40	1.20 (0.78, 1.84)	15	1.16 (0.58, 2.32)
≥70 years	246	52	1.21 (0.84, 1.75)	64	**1.41 (1.02, 1.95)**†	21	1.05 (0.63, 1.77)	8	1.19 (0.52, 2.77)
Histology									
Serous	758	176	**2.40 (1.39, 4.17)**†	220	**1.71 (1.18, 2.48)**†	96	**1.70 (1.05, 2.76)**†	34	**2.42 (1.08, 5.45)**†
Mucinous	115	5	1.46 (0.52, 4.07)	1	**0.10 (0.01, 0.74)**†	2	**0.22 (0.05, 0.94)**†	6	1.72 (0.60, 4.99)
Endometrioid	298	14	1.00 (ref)	34	1.00 (ref)	23	1.00 (ref)	8	1.00 (ref)
Clear cell	94	5	1.36 (0.49, 3.80)	10	1.28 (0.53, 2.60)	9	1.35 (0.62, 2.93)	1	0.39 (0.05, 3.16)
Other	129	24	**2.12 (1.09, 4.13)**†	28	1.33 (0.80, 2.21)	11	0.92 (0.44, 1.93)	6	1.92 (0.65, 5.70)
Stage									
I	254	0	1.00 (ref)	15	1.00 (ref)	146	1.00 (ref)	63	1.00 (ref)
II	247	4	-	21	**4.59 (1.57, 13.44)**†	197	**2.41 (1.10, 5.29)**†	71	**3.32 (1.17, 9.44)**†
III	699	9	-	22	**17.70 (6.44, 48.65)**‡	88	**5.56 (2.68, 11.53)**‡	22	**4.76 (1.74, 13.04)**†
IV	194	5	-	14	**30.08 (10.71, 84.51)**‡	33	**10.98 (4.84, 24.92)**‡	3	**4.37 (0.99, 19.25)**†
Residual disease									
No	300	14	1.00 (ref)	30	1.00 (ref)	18	1.00 (ref)	13	1.00 (ref)
Yes	533	141	**2.27 (1.29, 3.99)**†	166	**2.35 (1.57, 3.52)**‡	62	**2.31 (1.33, 4.02)**†	22	1.67 (0.79, 3.53)
**Reproductive/hormonal factors**									
Oral contraceptive use									
Never	568	92	1.00 (ref)	122	1.00 (ref)	60	1.00 (ref)	24	1.00 (ref)
Ever	732	90	0.83 (0.59, 1.15)	154	1.11 (0.85, 1.44)	79	0.99 (0.68, 1.44)	27	0.84 (0.45, 1.54)
Duration of oral contraceptive use (years)									
0	570	80	1.00 (ref)	131	1.00 (ref)	62	1.00 (ref)	22	1.00 (ref)
1-5	307	39	1.06 (0.71, 1.59)	70	1.10 (0.81, 1.48)	34	1.06 (0.69, 1.64)	12	1.18 (0.57, 2.44)
>5	219	28	1.03 (0.65, 1.64)	37	0.82 (0.56, 1.19)	24	0.87 (0.53, 1.44)	8	0.86 (0.37, 2.03)
Estrogen hormone replacement therapy									
Never	977	136	1.00 (ref)	200	1.00 (ref)	102	1.00 (ref)	33	1.00 (ref)
Ever	322	45	0.94 (0.66, 1.33)	76	0.89 (0.67, 1.17)	36	0.77 (0.51, 1.14)	18	1.46 (0.77, 2.76)
Endometriosis									
No	1219	166	1.00 (ref)	261	1.00 (ref)	132	1.00 (ref)	49	1.00 (ref)

Yes	70	9	1.13 (0.57, 2.23)	14	1.11 (0.64, 1.91)	6	0.95 (0.41, 2.18)	2	1.00 (0.24, 4.25)
Parity, never/ever									
Nulliparous	218	36	1.00 (ref)	32	1.00 (ref)	24	1.00 (ref)	6	1.00 (ref)
Parous	1084	147	0.73 (0.50, 1.05)	244	1.26 (0.87, 1.84)	115	0.90 (0.57, 1.42)	45	1.12 (0.46, 2.71)
Parity [b]									
1	190	20	1.00 (ref)	32	1.00 (ref)	22	1.00 (ref)	9	1.00 (ref)
2	407	56	1.26 (0.75, 2.10)	89	1.26 (0.84, 1.89)	39	0.96 (0.57, 1.63)	17	1.01 (0.45, 2.29)
>3	440	65	1.18 (0.71, 1.96)	114	1.39 (0.93, 2.09)	48	1.11 (0.66, 1.87)	16	0.87 (0.38, 2.01)
Age at first birth [b]									
<20	117	20	1.00 (ref)	24	1.00 (ref)	14	1.00 (ref)	6	1.00 (ref)
20-30	685	85	0.73 (0.45, 1.20)	154	1.09 (0.71, 1.69)	70	0.76 (0.43, 1.36)	30	0.72 (0.29, 1.77)
>30	107	10	**0.44 (0.20, 0.94)**[†]	33	1.53 (0.90, 2.60)	10	0.34 (0.37, 1.90)	3	0.72 (0.78, 2.94)
Breastfed [b]									
Never	434	51	1.00 (ref)	97	1.00 (ref)	46	1.00 (ref)	18	1.00 (ref)
Ever	603	90	1.38 (0.97, 1.96)	138	1.15 (0.88, 1.50)	63	1.09 (0.74, 1.60)	24	0.90 (0.48, 1.68)
Duration of breastfeeding (months) [b]									
0	427	51	1.00 (ref)	94	1.00 (ref)	44	1.00 (ref)	18	1.00 (ref)
1-6	286	44	1.45 (0.96, 2.20)	59	1.06 (0.76, 1.48)	31	1.12 (0.70, 1.79)	11	0.83 (0.39, 1.80)
>6	313	43	1.22 (0.81, 1.84)	79	1.24 (0.91, 1.68)	32	1.14 (0.72, 1.80)	13	0.97 (0.47, 1.99)
Age at menarche (years)									
≤ 12	489	79	1.00 (ref)	92	1.00 (ref)	49	1.00 (ref)	26	1.00 (ref)
13	409	44	0.71 (0.49, 1.03)	92	1.20 (0.71, 1.47)	43	1.11 (0.73, 1.68)	14	0.65 (0.34, 1.27)
14	221	32	0.73 (0.48, 1.12)	44	1.02 (0.71, 1.47)	25	1.21 (0.74, 1.98)	6	0.48 (1.20, 1.19)
>14	178	27	0.80 (0.51, 1.24)	46	1.38 (0.97, 1.98)	22	1.61 (0.97, 2.67)	5	0.75 (0.29, 1.96)
Age at natural menopause									
≤ 47	175	32	1.00 (ref)	39	1.00 (ref)	16	1.00 (ref)	4	1.00 (ref)
>47-50	228	30	0.84 (0.50, 1.40)	53	0.92 (0.60, 1.41)	30	1.24 (0.64, 2.38)	11	1.89 (0.85, 6.13)
>50-52	147	19	0.87 (0.49, 1.56)	40	1.18 (0.74, 1.86)	11	0.92 (0.41, 2.09)	4	1.09 (0.27, 4.50)
>52	142	17	0.65 (0.36, 1.20)	32	0.82 (0.50, 1.33)	17	1.10 (0.53, 2.30)	5	1.29 (0.34, 4.87)
Menopausal status at diagnosis									
Premenopausal	370	43	1.00 (ref)	61	1.00 (ref)	37	1.00 (ref)	17	1.00 (ref)
Postmenopausal	907	136	1.18 (0.69, 2.03)	210	1.34 (0.83, 2.16)	102	1.09 (0.59, 2.02)	34	0.59 (0.23, 1.53)
IUD use									
Never	1090	153	1.00 (ref)	227	1.00 (ref)	116	1.00 (ref)	45	1.00 (ref)
Ever	206	25	0.84 (0.54, 1.30)	48	1.06 (0.77, 1.46)	23	0.98 (0.62, 1.55)	6	0.59 (0.25, 1.40)
Number of ovulatory cycles									
≤322.28	228	26	1.00 (ref)	49	1.00 (ref)	19	1.00 (ref)	7	1.00 (ref)
>322.28 - 389.52	230	36	1.37 (0.80, 2.33)	52	0.89 (0.60, 1.33)	27	1.11 (0.60, 2.05)	13	1.99 (0.73, 5.39)

>389.52 - 439.42	225	27	1.03 (0.56, 1.90)	59	0.97 (0.64, 1.46)	26	0.12 (0.58, 2.15)	9	1.23 (0.38, 4.00)
>439.42	225	31	1.23 (0.67, 2.26)	48	0.87 (0.55, 1.36)	25	1.08 (0.54, 2.17)	9	1.31 (0.39, 4.38)
**Lifestyle factors/family history**									
Smoking, never/ever									
Never	661	96	1.00 (ref)	139	1.00 (ref)	64	1.00 (ref)	21	1.00 (ref)
Ever	640	86	0.93 (0.69, 1.24)	137	0.93 (0.74, 1.18)	75	1.17 (0.84, 1.63)	30	1.65 (0.94, 2.90)
BMI 5 years prior to diagnosis (kg/m^2)									
Underweight	20	6	1.70 (0.74, 3.95)	2	0.49 (0.12, 1.96)	3	1.08 (0.34, 3.45)	0	-
Normal	660	89	1.00 (ref)	138	1.00 (ref)	69	1.00 (ref)	25	1.00 (ref)
Overweight	388	53	1.12 (0.80, 1.57)	86	1.06 (0.81, 1.40)	44	1.18 (0.81, 1.73)	11	0.87 (0.43, 1.78)
Obese	226	31	1.24 (0.82, 1.87)	49	1.17 (0.85, 1.63)	23	1.08 (0.67, 1.74)	15	**2.15 (1.12, 4.10)**[†]

[a] All risk factors were adjusted for age at diagnosis, histology, and stage.

[b] Among parous women only.

[†] $P<0.05$

[‡] $P<0.001$

Table S2. HR and 95% CI of lifestyle factors and all-cause mortality by survival time intervals, additionally adjusted for residual disease [a]

	N	< 3 years		3 - < 6 years		6 - < 10 years		≥ 10 years	
		Cases	HR (95% CI)	Cases	HR (95% CI)	Cases	HR (95% CI)	Cases	HR (95% CI)
**SMOKING, NEVER/EVER**									
**Original**									
Never	661	104	1.00 (ref)	151	1.00 (ref)	80	1.00 (ref)	64	1.00 (ref)
Ever	640	90	0.89 (0.67, 1.18)	153	0.97 (0.77, 1.22)	89	1.12 (0.83, 1.52)	98	**1.75 (1.27, 2.40)**†
**Adjusted**									
Never	372	70	1.00 (ref)	90	1.00 (ref)	45	1.00 (ref)	38	1.00 (ref)
Ever	393	62	0.84 (0.60, 1.19)	107	1.07 (0.81, 1.43)	52	1.11 (0.74, 1.67)	62	**1.76 (1.17, 2.65)**†
**BMI 5 YEARS PRIOR TO DIAGNOSIS (KG/M^2)**									
**Original**									
Underweight	20	6	1.69 (0.73, 3.90)	2	0.46 (0.11, 1.85)	3	0.99 (0.31, 3.17)	1	0.47 (0.07, 3.38)
Normal	660	94	1.00 (ref)	155	1.00 (ref)	81	1.00 (ref)	81	1.00 (ref)
Overweight	388	56	1.12 (0.80, 1.56)	95	1.04 (0.80, 1.34)	58	1.32 (0.94, 1.86)	39	0.84 (0.57, 1.24)
Obese	226	35	1.31 (0.89, 1.94)	51	1.09 (0.80, 1.50)	27	1.09 (0.70, 1.70)	41	**1.81 (1.24, 2.65)**†
**Adjusted**									
Underweight	9	2	1.25 (0.30, 5.20)	1	0.33 (0.05, 2.40)	3	1.97 (0.60, 6.46)	0	-
Normal	383	62	1.00 (ref)	200	1.00 (ref)	42	1.00 (ref)	50	1.00 (ref)
Overweight	229	37	1.17 (0.77, 1.76)	61	0.96 (0.70, 1.33)	38	**1.82 (1.16, 2.86)**†	22	0.99 (0.58, 1.67)
Obese	137	27	1.54 (0.98, 2.44)	34	1.07 (0.73, 1.59)	14	1.22 (0.67, 2.25)	28	**2.56 (1.58, 4.15)** ‡

[a] All risk factors were adjusted for age at diagnosis, histology, stage, and residual disease.

†$P<0.05$

‡P<0.0001

Table S3. HR and 95% CI of lifestyle factors and all-cause mortality by survival time intervals, stratified by stage at diagnosis [a]

	N	< 3 years		3 - < 6 years		6 - < 10 years		≥ 10 years	
		**Cases**	**HR (95% CI)**	**Cases**	**HR (95% CI)**	**Cases**	**HR (95% CI)**	**Cases**	**HR (95% CI)**
**SMOKING, NEVER/EVER**									
**Overall**									
Never	661	104	1.00 (ref)	151	1.00 (ref)	80	1.00 (ref)	64	1.00 (ref)
Ever	640	90	0.89 (0.67, 1.18)	153	0.97 (0.77, 1.22)	89	1.12 (0.83, 1.52)	98	**1.75 (1.27, 2.40)**†
**Stage I + II**									
Never	252	5	1.00 (ref)	15	1.00 (ref)	27	1.00 (ref)	36	1.00 (ref)
Ever	226	9	1.71 (0.57, 5.09)	15	1.20 (0.59, 2.47)	15	0.68 (0.36, 1.27)	48	1.52 (0.98, 2.36)
**Stage III + IV**									
Never	409	99	1.00 (ref)	136	1.00 (ref)	53	1.00 (ref)	28	1.00 (ref)
Ever	414	81	0.83 (0.62, 1.11)	138	0.94 (0.74, 1.19)	74	1.30 (0.91, 1.86)	50	**1.98 (1.24, 3.15)**†
**BMI 5 YEARS PRIOR TO DIAGNOSIS (KG/M^2)**									
**Overall**									
Underweight	20	6	1.69 (0.73, 3.90)	2	0.46 (0.11, 1.85)	3	0.99 (0.31, 3.17)	1	0.47 (0.07, 3.38)
Normal	660	94	1.00 (ref)	155	1.00 (ref)	81	1.00 (ref)	81	1.00 (ref)
Overweight	388	56	1.12 (0.80, 1.56)	95	1.04 (0.80, 1.34)	58	1.32 (0.94, 1.86)	39	0.84 (0.57, 1.24)
Obese	226	35	1.31 (0.89, 1.94)	51	1.09 (0.80, 1.50)	27	1.09 (0.70, 1.70)	41	**1.81 (1.24, 2.65)**†
**Stage I + II**									
Underweight	6	0	-	0	-	1	2.24 (0.30, 16.76)	0	-
Normal	248	6	1.00 (ref)	18	1.00 (ref)	21	1.00 (ref)	43	1.00 (ref)
Overweight	140	7	2.21 (0.74, 6.60)	8	0.88 (0.38, 2.03)	13	1.18 (0.59, 2.36)	21	0.84 (0.50, 1.42)
Obese	82	1	0.56 (0.07, 4.65)	4	0.69 (0.23, 2.05)	7	1.02 (0.43, 2.41)	20	1.52 (0.89, 2.61)
**Stage III + IV**									
Underweight	14	6	1.82 (0.79, 4.20)	2	0.51 (0.13, 2.05)	2	0.85 (0.21, 3.49)	1	0.68 (0.09, 4.97)
Normal	412	88	1.00 (ref)	137	1.00 (ref)	60	1.00 (ref)	38	1.00 (ref)
Overweight	248	49	1.04 (0.73, 1.47)	87	1.04 (0.79, 1.36)	45	1.37 (0.93, 2.02)	18	0.83 (0.47, 1.47)
Obese	144	34	1.31 (0.88, 1.96)	47	1.12 (0.81, 1.56)	20	1.13 (0.68, 1.88)	21	**2.17 (1.27, 3.70)** †

[a] All risk factors were adjusted for age at diagnosis and histology.

† $P<0.05$

Table S4. HR and 95% CI of risk factors and all-cause mortality by survival time intervals among HGSC ovarian cancer cases (N=688) [a,b]

	N	< 3 years		3 - < 6 years		6 - < 10 years		≥ 10 years	
		Cases	HR (95% CI)	Cases	HR (95% CI)	Cases	HR (95% CI)	Cases	HR (95% CI)
**Tumour characteristics**									
Age at diagnosis, n (%)									
<50 years	143	34	0.97 (0.63, 1.50)	42	1.05 (0.71, 1.56)	18	0.85 (0.47, 1.52)	11	0.69 (0.32, 1.45)
50 to <60 years	202	49	1.00 (ref)	62	1.00 (ref)	31	1.00 (ref)	18	1.00 (ref)
60 to <70 years	200	51	1.23 (0.83, 1.82)	68	1.36 (0.96, 1.92)	33	1.36 (0.83, 2.22)	21	**1.87 (0.99, 3.53)** †
≥70 years	135	38	1.18 (0.77, 1.80)	49	**1.49 (1.03, 2.17)**†	20	1.47 (0.84, 2.59)	18	**3.34 (1.72, 6.47)**‡
Stage									
I	15	0	1.00 (ref)	0	1.00 (ref)	2	1.00 (ref)	3	1.00 (ref)
II	67	8	-	11	-	9	1.44 (0.31, 6.68)	14	1.31 (0.37, 4.58)
III	449	109	-	147	-	70	3.39 (0.83, 13.91)	45	1.63 (0.50, 5.27)
IV	149	55	-	63	-	21	**9.06 (2.11, 38.97)** †	6	**4.21 (1.03, 17.17)** †
Residual disease									
No	70	7	1.00 (ref)	17	1.00 (ref)	12	1.00 (ref)	10	1.00 (ref)
Yes	342	114	**2.55 (1.19, 5.47)** †	1210	**2.04 (1.22, 3.40)** †	42	1.27 (0.67, 2.42)	33	1.67 (0.82, 3.41)
**Reproductive/hormonal factors**									
Breastfed [c]									
Never	220	43	1.00 (ref)	83	1.00 (ref)	36	1.00 (ref)	28	1.00 (ref)
Ever	305	67	1.26 (0.85, 1.86)	99	0.90 (0.67, 1.21)	46	0.93 (0.60, 1.45)	24	**0.55 (0.32, 0.95)** †
Duration of breastfeeding (months) [c]									
0	217	43	1.00 (ref)	82	1.00 (ref)	34	1.00 (ref)	28	1.00 (ref)
1-6	142	32	1.29 (0.81, 2.05)	40	0.77 (0.52, 1.13)	23	0.98 (0.57, 1.67)	14	0.67 (0.35, 1.27)
>6	161	33	1.13 (0.71, 1.79)	59	0.99 (0.71, 1.39)	23	0.97 (0.57, 1.65)	10	**0.43 (0.21, 0.90)** †
Age at menarche (years)									
≤ 12	230	60	1.00 (ref)	63	1.00 (ref)	35	1.00 (ref)	26	1.00 (ref)
13	194	30	**0.59 (0.38, 0.92)** †	74	1.22 (0.87, 1.71)	32	1.02 (0.63, 1.66)	23	0.99 (0.56, 1.74)
14	112	24	0.70 (0.44, 1.13)	37	1.15 (0.76, 1.73)	21	1.39 (0.80, 2.41)	8	0.64 (0.29, 1.41)
>14	89	24	0.86 (0.53, 1.39)	35	1.41 (0.93, 2.14)	14	1.40 (0.75, 2.62)	6	0.83 (0.34, 2.02)
**Lifestyle factors**									
Smoking, never/ever									
Never	319	72	1.00 (ref)	109	1.00 (ref)	48	1.00 (ref)	22	1.00 (ref)
Ever	308	66	0.95 (0.68, 1.33)	101	0.85 (0.64, 1.11)	54	1.09 (0.74, 1.61)	41	**2.33 (1.38, 3.93)** †
BMI 5 years prior to diagnosis (kg/m^2)									
Underweight	11	4	1.23 (0.44, 3.39)	1	0.30 (0.04, 2.12)	3	1.19 (0.37, 3.85)	1	0.84 (0.11, 6.25)

Normal	325	71	1.00 (ref)	103	1.00 (ref)	49	1.00 (ref)	36	1.00 (ref)
Overweight	180	36	0.95 (0.63, 1.42)	64	1.05 (0.77, 1.44)	34	1.31 (0.84, 2.03)	16	0.94 (0.52, 1.71)
Obese	106	24	1.23 (0.77, 1.96)	41	**1.47 (1.02, 2.11)** †	16	1.33 (0.75, 2.36)	10	1.34 (0.66, 2.71)

[a] All risk factors were adjusted for age at diagnosis and stage.

[b] High-grade serous carcinoma defined as tumors with serous histology and grade 2/3, or if grade missing stage 3/4.

[c] Among parous women only.

† $P<0.05$

‡ $P<0.001$
